# A Rare Case of Multifocal Cystadenomatous Oncocytic Hyperplasia of the Parotid Gland

**DOI:** 10.7759/cureus.45614

**Published:** 2023-09-20

**Authors:** Abdalrahman Albader, Nawaf Almotairi, Mohammad Alotaibi, Mishal M AlMutairi

**Affiliations:** 1 Otorhinolaryngology - Head and Neck Surgery, Kuwait Institute for Medical Specializations, Kuwait, KWT; 2 Otorhinolaryngology - Head and Neck Surgery, Farwaniya Hospital, Farwaniya, KWT

**Keywords:** oncocytoma, oncocytes, oncocytosis, parotid, multifocal adenomatous oncocytic hyperplasia

## Abstract

Multifocal cystadenomatous oncocytic hyperplasia of the parotid gland is an extremely rare non-neoplastic lesion. We present a case involving a 73-year-old female with a painless, small mass in her right preauricular region for the past two months. Fine needle aspiration suggested a diagnosis of mucoepidermoid carcinoma. Following further investigations, the patient underwent an uneventful right superficial parotidectomy without neck dissection. Histopathological examination of the excised superficial parotid tissue revealed multifocal cystadenomatous oncocytic hyperplasia with negative surrounding margins. The patient’s subsequent follow-ups in the outpatient department were satisfactory, with no reported issues, concerns, or evidence of recurrence.

## Introduction

Oncocytes are the main cells in oncocytic salivary gland lesions [1]. These cells are distinguished by their centrally positioned nucleus, characterized by condensed chromatin, and excessively packed with mitochondria that give rise to an eosinophilic, granular appearance in the cytoplasm. According to Lee et al., oncocytic lesions were first described by Shaffer in 1897 and further characterized by Hamperl in 1931 [2]. These lesions are rare, accounting for less than 1% of salivary gland tumors [1].

According to the World Health Organization classification, oncocytic lesions are classified into three types: oncocytosis, oncocytoma, and oncocytic carcinoma [3]. Oncocytes are present in various salivary gland lesions, making the cytological diagnosis of oncocytic lesions challenging and necessitating further histological examination [4]. Oncocytosis is a rare non-neoplastic lesion classified as diffuse oncocytosis and multifocal adenomatous oncocytic hyperplasia (MOAH) [5]. Oncocytoma is a salivary gland tumor primarily composed of oncocytes, sharing characteristic similarities with adenolymphoma (Warthin’s tumor), except that oncocytoma lacks lymphoid tissue [1]. Oncocytoma can be found in various organs, such as salivary glands, endocrine glands, kidneys, breasts, testes, buccal mucosa, upper respiratory tract, and ocular adnexa, and demonstrates similar histological features when arising in different sites of the body [1].

MAOH is a rare pathological condition of parotid neoplasms, characterized by oncocytic proliferation of the parotid gland’s duct system [1]. In this case study, we report a case of unilateral multifocal cystadenomatous oncocytic hyperplasia of the parotid gland, discussing its diagnosis and management. This case report has been prepared per the CARE criteria for case reports [6].

## Case presentation

**Figure 1:**
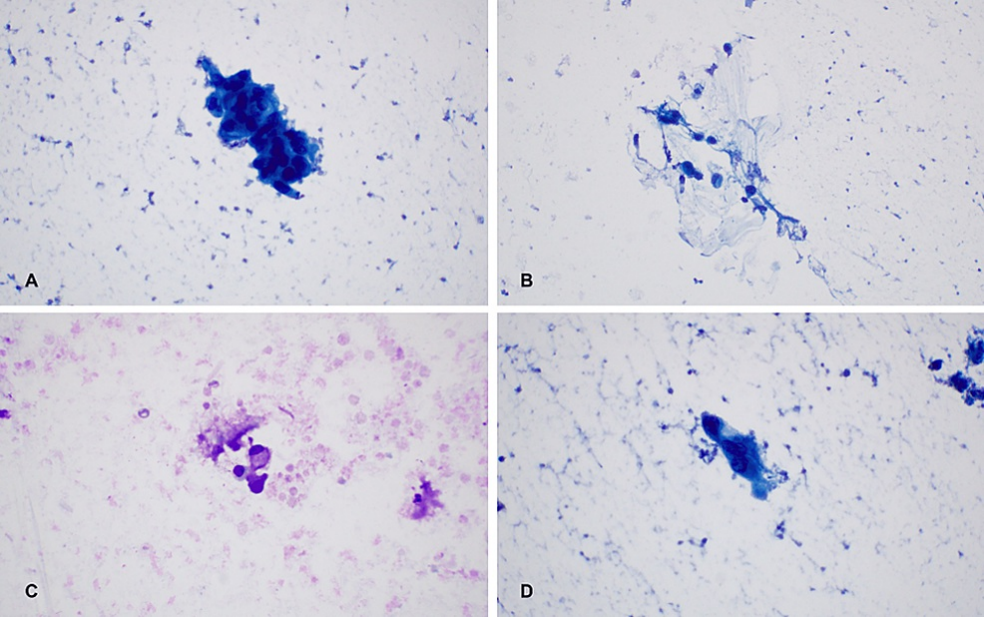
Morphological features of tumor cells and mucin secretion. A. (60x Papanicolaou stain) columnar atypical tumor cells. B. (40x Papanicolaou stain) tumor cells embedded in mucin. C. (60x DQ1 stain) mucin secretion in tumor cells. D. (60x Papanicolaou stain) malignant cellular morphology

A 73-year-old female with a past medical history of type 2 diabetes presented to the outpatient clinic in the Department of Otorhinolaryngology - Head and Neck Surgery at Farwaniya Hospital in Kuwait with a small, asymptomatic, non-painful preauricular mass on the right side present for two months. A comprehensive head and neck examination, including lymph node palpation, revealed nothing remarkable except for a mass measuring 2.5 centimeters. She had initially undergone fine needle aspiration cytology (FNAC) for this mass, the results of which suggested mucoepidermoid carcinoma (Figure 1).

**Figure 2:**
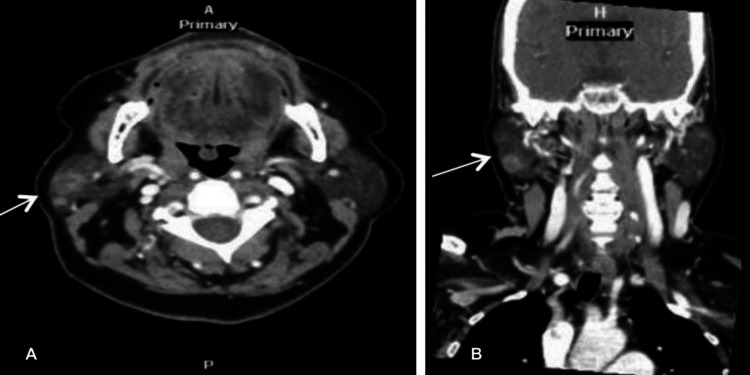
CT neck imaging of the parotid gland lesion. Axial (A) and coronal (B) views of a contrast-enhanced CT neck scan revealing a well-defined, non-homogeneous lesion in the right parotid gland (white arrows) measuring approximately 13 x 12 x 10.5 mm. No surrounding tissue infiltration is observed

She underwent a contrasted CT scan of the neck, which showed a well-defined, non-homogenous lesion of the right parotid gland with no infiltration of the surrounding right parotid tissue (Figure 2). Additionally, a CT thoracic scan was conducted, resulting in unremarkable findings.

A fluorodeoxyglucose positron emission tomography scan was also performed and found unremarkable for metastasis.

**Figure 3:**
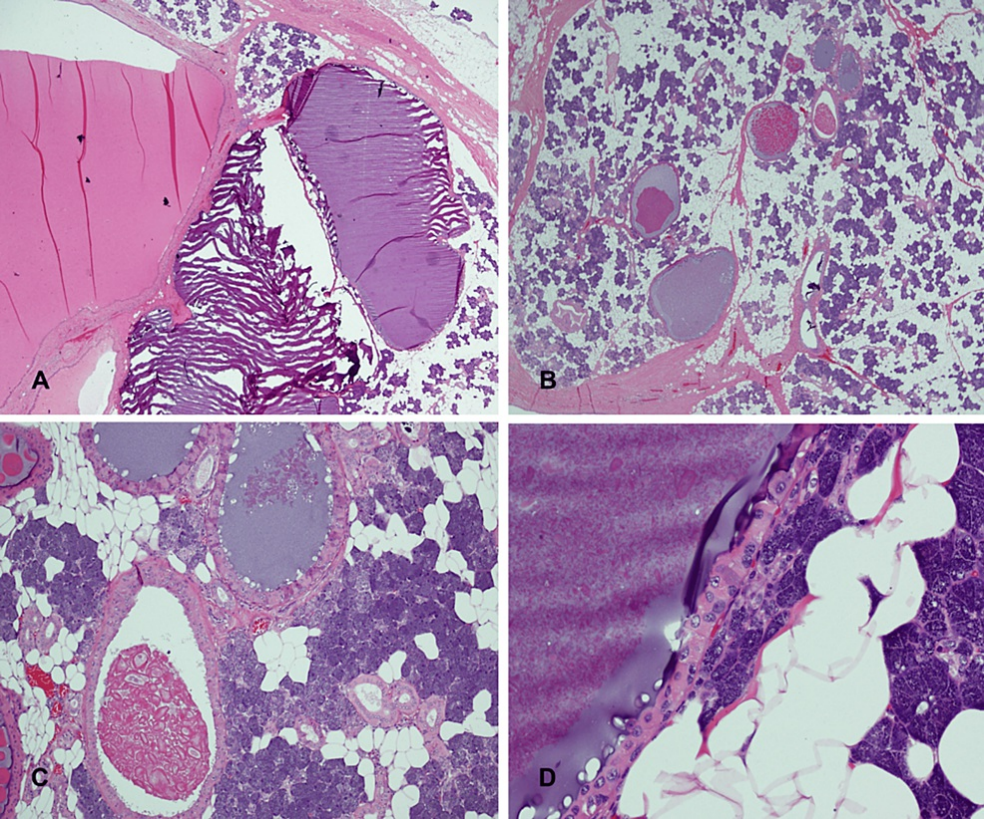
Histopathological characteristics of intraparotid cysts. A. (H&E stain, x20) intraparotid cystic spaces/ducts with basophilic to amphophilic secretions. B. (H&E stain, x20) multiple intraparotid cysts of varying sizes. C. (H&E stain, x100) cysts lined by stratified ductal cells with oncocytic hyperplasia, containing basophilic secretions, diverse crystalloids, and calcification. D. (H&E stain, x400) oncocytic metaplasia in cyst epithelial lining, characterized by large, polygonal cells with granular, intensely acidophilic cytoplasm, round uniform nuclei, distinct nucleoli, and low nucleus-cytoplasm ratio

Results from her routine laboratory investigations, including full blood count, renal function test, and coagulation profile, were within reference ranges. The patient was admitted and consented to right superficial parotidectomy. The operation also included taking specimens from the anterior, posterior, inferior, superior, and deep margins, a right deep lobe specimen, and an upper cervical lymph node. All specimens were sent for histopathology evaluation and returned negative results. The histopathology report of the right superficial parotid gland revealed multifocal cystadenomatous oncocytic hyperplasia (Figure 3).

Postoperatively, the patient had an uneventful three-day hospital stay and was subsequently discharged. Her frequent six-month outpatient clinic visits were satisfactory, with no new issues or concerns reported.

## Discussion

In this case report, we presented a 73-year-old female patient diagnosed with the rare condition of MAOH of the parotid gland. Oncocytic transformation results from metaplastic development in reaction to detrimental alterations occurring within the cells or due to the natural aging process. These changes result in the depletion of functional mitochondrial enzymes, prompting a compensatory response characterized by the proliferation of mitochondria. This increased mitochondrial presence is accountable for the distinctive microscopic alterations observed in oncocytic cells [7].

FNAC might not accurately identify whether an oncocytic neoplasm is benign or malignant [8]. In our case, mucoepidermoid carcinoma was initially identified through FNAC, but histological examination later diagnosed the condition as MAOH. Shellenberger et al. found that oncocytic metaplastic nodules demonstrate an inhomogeneous mild degree of enhancement on CT findings, whereas oncocytoma nodules show a well-defined mildly enhancing tumor with compression of the surrounding parenchyma [9]. Synchronous oncocytosis and oncocytoma in a single parotid specimen have been reported by Shellen et al. and Capone et al. [9,10].

The standard and cornerstone treatment for MAOH is parotidectomy with facial nerve sparing. Patients should be followed up frequently for any local recurrences arising from residual tumors in case of incomplete resection. A peripheral residual clearance of about 5 mm should be performed during such procedures [7]. Parotidectomy can be either superficial or total [10].

## Conclusions

In conclusion, we report a rare case of MAOH in the parotid gland, highlighting the diagnostic challenges due to its similarities with various benign and malignant neoplastic lesions. This case emphasizes cytology's limitations and histopathology's importance for establishing a definitive diagnosis. The standard treatment for MAOH is parotidectomy with facial nerve sparing, and careful follow-up is crucial for monitoring local recurrences. Further studies are needed to determine this condition's exact pathophysiology, risk factors, and prevalence.
